# Methylation and Demethylation of Emerging Contaminants Changed Bioaccumulation and Acute Toxicity in **Daphnia magna**

**DOI:** 10.1021/acs.est.3c03242

**Published:** 2023-09-28

**Authors:** Yaxin Xiong, Qingyang Shi, Aspen Smith, Daniel Schlenk, Jay Gan

**Affiliations:** Department of Environmental Sciences, University of California, Riverside, California 92521, United States

**Keywords:** CECs, methylation/demethylation, interconversion, transformation products, aquatic invertebrates, *Daphnia magna*

## Abstract

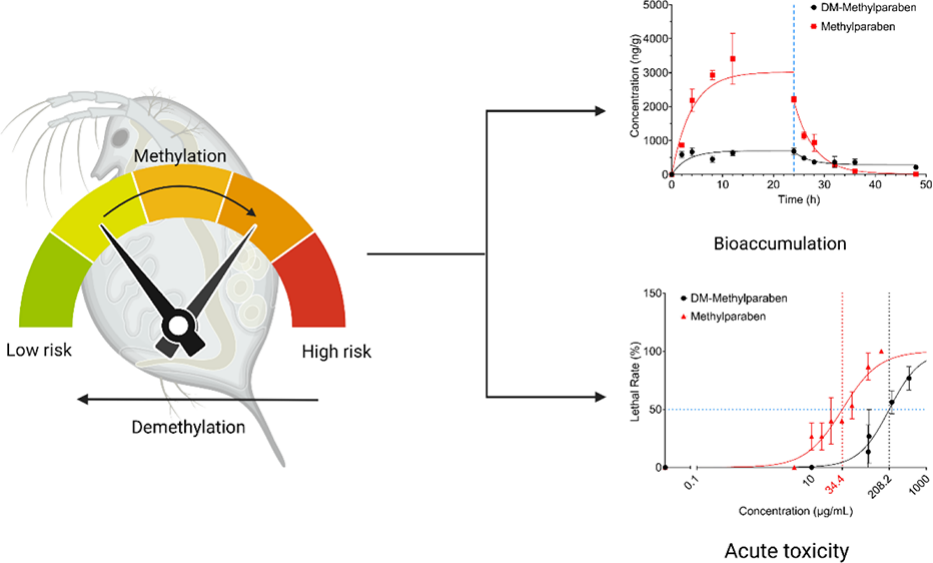

Contaminants of emerging concern (CECs) in the environment undergo various transformations, leading to the formation of transformation products (TPs) with a modified ecological risk potential. Although the environmental significance of TPs is increasingly recognized, there has been relatively little research to understand the influences of such transformations on subsequent ecotoxicological safety. In this study, we used four pairs of CECs and their methylated or demethylated derivatives as examples to characterize changes in bioaccumulation and acute toxicity in **Daphnia magna**, as a result of methylation or demethylation. The experimental results were further compared to quantitative structure–activity relationship (QSAR) predictions. The methylated counterpart in each pair generally showed greater acute toxicity in **D. magna**, which was attributed to their increased hydrophobicity. For example, the LC_50_ values of methylparaben (34.4 ± 4.3 mg L^–1^) and its demethylated product (225.6 ± 17.3 mg L^–1^) differed about eightfold in **D. magna**. The methylated derivative generally exhibited greater bioaccumulation than the demethylated counterpart. For instance, the bioaccumulation of methylated acetaminophen was about 33-fold greater than that of acetaminophen. *In silico* predictions via QSARs aligned well with the experimental results and suggested an increased persistence of the methylated forms. The study findings underline the consequences of simple changes in chemical structures induced by transformations such as methylation and demethylation and highlight the need to consider TPs to achieve a more holistic understanding of the environmental fate and risks of CECs.

## Abstract 2

Common transformations of CECs such as methylation and demethylation may cause changes in acute toxicity and bioaccumulation in aquatic organisms, with close dependence on chemical structures.

## 1 Introduction

The occurrence of numerous contaminants of emerging concern (CECs) in the effluent from wastewater treatment plants (WWTPs) and impacted aquatic environments has been extensively reported.^1−4^ Many CECs contain reactive functional groups, such as hydroxyl, carboxyl, and amide groups, making them susceptible to various biotic and abiotic transformation reactions.^5−8^ However, most research has focused on the parent form of CECs while generally neglecting their transformation products (TPs) that are often in coexistence. Transformations, such as methylation and demethylation, have been observed in various environmental matrices for many CECs.^6,9−14^ For example, previous studies showed the co-occurrence of methylated TPs of triclosan, bisphenol A (BPA), tetrabromobisphenol A (TBBPA), and acetaminophen under biotic and/or abiotic conditions with their parent forms.^7,13,15−17^ On the other hand, demethylation is a major metabolism pathway for CECs in biological systems, as observed for CECs such as naproxen and diazepam in humans.^18,19^

Despite the fact that TPs seem to occur readily and coexist with their parent form in the environment, the ecotoxicological consequences of such transformations have not been adequately considered. By inducing changes in their chemical structures, such as the addition or loss of a methyl group caused by methylation or demethylation, transformations can significantly change a compound’s physicochemical properties.^20^ Hence, the ecotoxicological consequences of transformation products (TPs) may be subsequently altered. Moreover, changes in ecotoxicological profiles induced by the same transformation may be specific to different chemical structures. For instance, methylated products of diclofenac, BPA, triclosan, and 6-OH-BDE-47 all displayed enhanced toxicity or bioaccumulation potential in aquatic organisms, likely due to their increased hydrophobicity (log *K*_ow_) after methylation.^6,14−16,21^ However, methylated TBBPA ethers showed significantly lower acute toxicity in earthworms as compared to TBBPA, despite of increases in their log *K*_ow_ values.^22^ To date, ecotoxicological consequences as a result of methylation or demethylation have yet to be systematically evaluated.

In this study, we comparatively explored the behaviors of four common CECs, i.e., acetaminophen, diazepam, methylparaben, and naproxen, and their methylated or demethylated TPs (i.e., *M*-acetaminophen, *DM*-diazepam, *DM*-methylparaben, and *DM*-naproxen) in **Daphnia magna** by considering their bioaccumulation, acute toxicity, and interconversions. These four pairs of compounds were chosen due to their wide use and ubiquitous detections in the environment. *DM*-Diazepam and *DM*-methylparaben are not only demethylated TPs but also a pharmaceutical and the raw industrial material for producing parabens, respectively. Quantitative structure–activity relationship (QSAR) models were further developed and used to describe the experimental results. The study findings highlight the importance of simple changes in the chemical structure as the result of transformations, such as methylation and demethylation, in understanding the overall environmental risks posed by CECs in aquatic environments.

## 2 Materials and Methods

### 2.1 Chemicals and Materials

The analytical standards (purity >98%) of the four pairs of compounds considered in this study were purchased from Sigma-Aldrich (St. Louis, Missouri), Santa Cruz (Dallas, Texas), or Toronto Research Chemicals (Toronto, Ontario, Canada). Their physicochemical properties are summarized in Table 1. Deuterated compounds *d*_4_-acetaminophen, *d*_5_-diazepam, *d*_4_-methylparaben, and *d*_3_-naproxen were purchased from Sigma-Aldrich, Toronto Research Center, or C/D/N isotopes (Pointe-Claire, Quebec, Canada) and used as internal standards. HPLC-grade methanol was purchased from Fisher Scientific (Fair Lawn, New Jersey). Ultrapure water was generated in-house using a Milli-Q water purification system (Millipore, Carrigtwohill, Cork, Ireland).

**Table 1:**
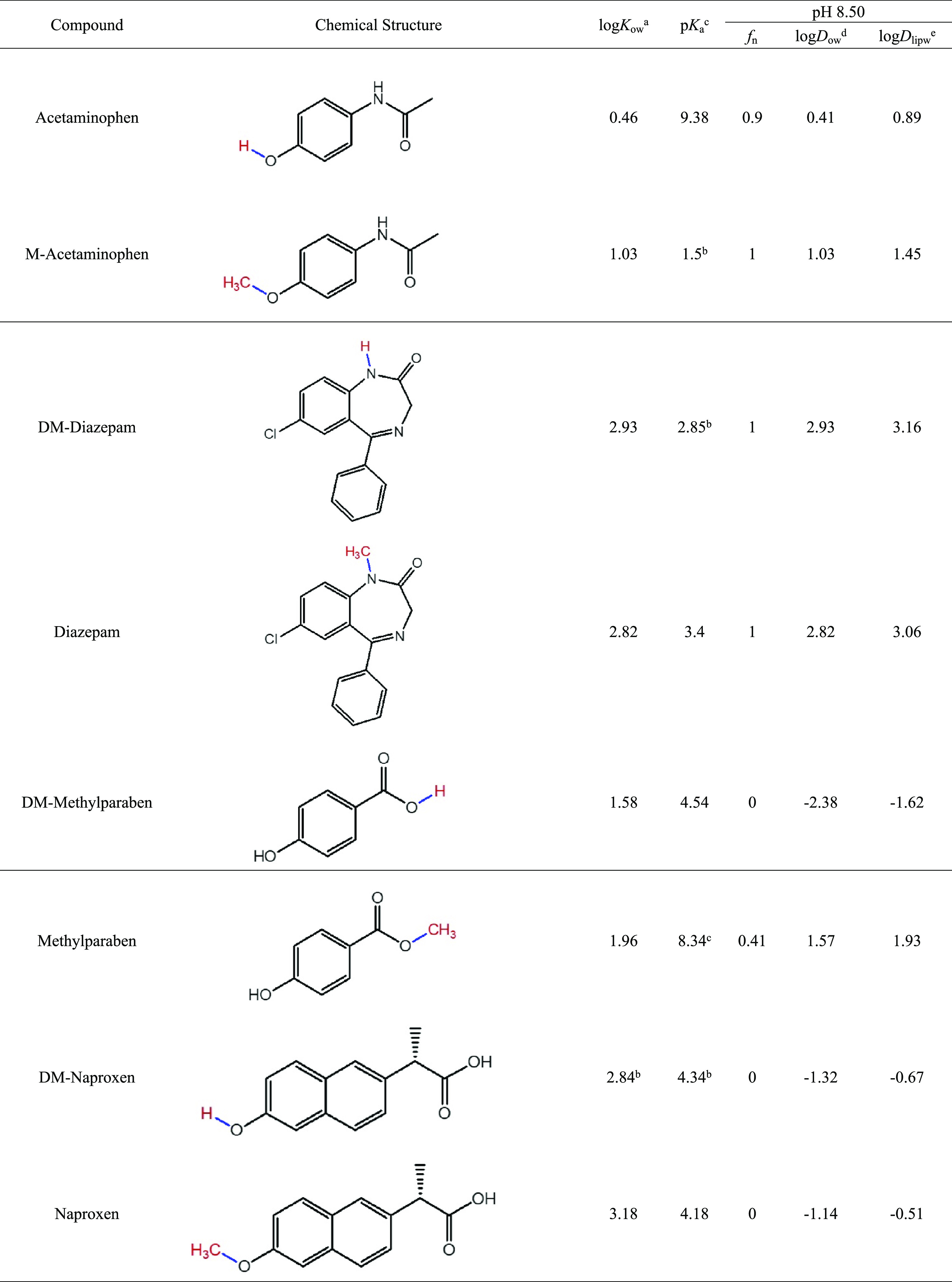
Physicochemical Properties of Selected CECs and Their Methylation/Demethylation Counterparts

a Measured values from PubChem: https://PubChem.ncbi.nlm.nih.gov/.

b Predicted by ChemAxon or retrieved from The Human Metabolome Database: https://hmdb.ca/.

c Measured value from CompTox Chemicals Dashboard: https://CompTox.epa.gov/dashboard/chemical/properties/DTXSID4022529.

d Calculated log*D*_ow_ values crosschecked with the log*D*_ow_ values predicted by ChemAxon: https://disco.chemaxon.com/calculators/demo/plugins/logd/.

The pH of the test medium in this study was measured to be 8.50 ± 0.10. The neutral fraction (*f*_n_) of the target compounds, their pH-adjusted octanol–water coefficients (log *D*_ow_), and their pH-adjusted lipid–water coefficients (log *D*_lipw_) were calculated for this pH condition (Table 1). The calculation and related details are given in the Supporting Information (SI) in Text S1.

**D. magna** was purchased from Aquatic Research Organisms (Hampton, New Hampshire) and maintained following the OECD Guidelines.^23^ Briefly, **D. magna** was raised in artificial freshwater (AFW) made by adding 58.5 mg of CaCl_2_·2H_2_O, 24.7 mg of MgSO_4_·7H_2_O, 13.0 mg of NaHCO_3_, and 1.2 mg of KCl into 1 L of deionized water. **D. magna** in AFW was maintained in a growth chamber at 21 °C with a 16:8 h (light:dark) photoperiod and fed daily with freshwater green algae (**Raphidocells subcapitata**). The medium was renewed twice a week to maintain a clear environment for the daphnids.

### 2.2 *D. magna* Acute Toxicity Tests

Acute toxicity testing of the target CECs and their derivatives to **D. magna** was carried out following the OECD Guidelines 202.^23^ According to the guideline, the immobilization rate of **D. magna** after 48 h of exposure was used to estimate the acute toxicity of the test compounds. Preliminary tests were conducted for each target compound at widely spaced concentrations to identify the concentration range for deriving an accurate dose–response curve. The stock solutions of individual test compounds were prepared in methanol and diluted with 10 mL of aerated AFW to different concentrations in 20 mL glass vials. The methanol volume was set to 100 μL in each vial as the solvent carrier, and a control group containing 100 μL of methanol without the target compound was used as the carrier solvent control. Each test included at least five concentration levels, and the concentrations of test compounds in the medium were experimentally measured (SI, Table S1). Each treatment included four replicates. Five **D. magna**, <24 h old at the beginning of the test and not the first brood progeny, were placed in each vial and maintained in the growth chamber under the same conditions as given above. Death of **D. magna** was determined by observing the lack of movement for 15 s after gentle agitation of the test vial, and the lethal rate of **D. magna** in each test vessel was recorded at 0, 24, and 48 h. The obtained dose–response data were fitted to the Boltzmann equation:^24^
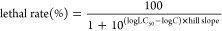
1where *C* is the measured concentration of the test compound. LC_50_ values at 48 h of the tested compounds were obtained using this equation.

### 2.3 *D. magna* Bioaccumulation Experiments

The bioaccumulation experiments were conducted in 500 mL glass beakers and consisted of a 24 h uptake phase and a 24 h depuration phase. The 250 mL of AFW was spiked with 0.25 mL of individual stock solution (1000 mg L^–1^ in methanol) at a nominal concentration of 1 mg L^–1^, and triplicates were used for each target compound. This high concentration level was chosen to facilitate the investigation of the potential interconversions between target CECs and their methylated or demethylated derivatives. For each beaker, 120 adult **D. magna** (21 days old) were added, and at 0, 2, 4, 8, 12, and 24 h, an aliquot of 10 **D. magna** and 1 mL of the test medium were withdrawn. After 24 h, the remaining **D. magna** was transferred to clean AFW to start the depuration phase. Similar to the uptake phase, 10 **D. magna** were removed from each beaker at 2, 4, 8, 12, and 24 h. The wet weight of each **D. magna** sample was recorded, and the samples were stored at −80 °C prior to chemical analysis.

The depuration data were fitted to a first-order decay model to obtain the depuration rate constant (*k*_d_, h^–1^):^25^
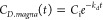
2where *C*_*D*. *magna*_ (μg kg^–1^, wet weight, w.w.) is the internal concentration of the target compound in **D. magna** and *C*_i_ (μg kg^–1^, w.w.) is the initial concentration of the target compound in **D. magna** when the depuration phase started, which is also the concentration when the uptake phase ended at 24 h. During the uptake phase, the concentration of the target compound in **D. magna** could be expressed as^25^
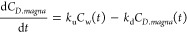
3where *k*_u_ is the uptake rate constant (L kg^–1^ h^–1^) and *C*_w_(*t*) is the concentration of the target compound (mg L^–1^) in water. Since *C*_*D*. *magna*_ at 0 h is zero and *C*_w_ is constant during the experiment, eq 3 can be simplified to eq 4 to estimate *k*_u_:^25^
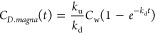
4

The dynamic bioconcentration factor (BCF, L kg^–1^, w.w.) may be further calculated by the following relationship:^25^
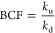
5

Along with the dynamic BCF, steady-state BCF (L kg^–1^, w.w.) was also calculated using the following equation:
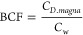
6where *C*_*D*. *magna*_ is the concentration of a target compound (μg kg^–1^, w.w.) in *D. magna* at equilibrium, which was 24 h in this study (Figure 2).

### 2.4 Sample Preparation and Instrument Analysis

Deuterated compounds were used as recovery surrogates. Before sample extraction, 10 μL of the stock solution containing the deuterated standard at 10 mg L^–1^ (in methanol) was added to the daphnid sample and the samples were extracted by sonication in 1 mL of methanol, followed by centrifugation at 14,000 rpm for 15 min. The same extraction was repeated for a total of three consecutive times, and the extracts were combined. The solvent extract was dried under nitrogen, recovered with 200 μL of water:methanol (1:1, v/v), and centrifuged at 14,000 rpm for 15 min. An aliquot of 100 μL of the cleaned extract was transferred to a 250 μL glass insert in a 2 mL LC vial for analysis on UPLC-MS/MS.

To obtain the actual concentration of target compounds in the aqueous medium, 50 μL of the deuterated mixture was added to 1 mL of solution sample in a 2 mL centrifuge tube, followed by 15 mg of Cleanert PEP powder (70–90 μm, Agela Technologies, Torrance, California). Samples were shaken by hand for 30 s and then subjected to vortexing for 1 min. The centrifuge tubes were centrifuged at 14,000 rpm for 15 min, and the liquid phase was discarded. The remaining solid powder was subjected to the same extraction step with 1 mL of methanol. The extract after centrifugation was transferred to another 2 mL centrifuge tube, dried under nitrogen, reconstituted in 1 mL of water:methanol (1:1, v/v), and filtered through a 0.2 μm PTFE filter into a 2 mL glass LC vial for instrument analysis.

Instrument analysis of all compounds was carried out on a Waters ACQUITY TQD ultraperformance liquid chromatography-tandem quadrupole mass spectrometry (UPLC-MS/MS) (Waters, Milford, Massachusetts). Chromatographic separation was performed at 40 °C using an ACQUITY BEH C18 column (100 × 2.1 mm inner diameter, 1.7 μm; Waters, Milford, Massachusetts). Mobile phase A was 0.01% formic acid in water (v/v), and mobile phase B was methanol. The mobile phases were programmed to the following gradient (with respect to mobile phase B): 0–1 min, 5–40%; 1–2 min, 40–90%; 2–4 min, 90–95%; and 4–6 min, 95–5%. The flow rate was maintained at 0.3 mL/min. The MRM transitions of all tested compounds were optimized and are listed in Table S2. Data were processed using TargetLynx XS software (Waters, Milford, Massachusetts).

### 2.5 *In Silico* Predictions

To better understand the effects of methylation and demethylation on the environmental behaviors of CECs, *in silico* predictions were made using QSAR models for acute toxicity, bioaccumulation, and persistence of the test compounds. The QSAR models computed the environmental behaviors of chemicals based on their chemical structures and available experimental data sets of compounds of similar structures. The consensus QSAR method in the U.S. EPA’s Toxicity Estimation Software Tool (T.E.S.T., version 5.1.2) was used to predict the acute toxicity of the target compounds in **D. magna**.^26^ The BCF and the biotransformation half-life values of test compounds were obtained by using the BCFBAF model in the U.S. EPA’s EPI Suite software (version 4.11). As a similar model is not available for aquatic invertebrates, *in silico* BCF values for lower trophic fish were predicted using the Arnot–Gobas method as an approximation for **D. magna**.^27^ Similarly, *in silico* half-life values were derived from the estimated whole-body primary biotransformation rate in fish and normalized to a 10 g fish at 15 °C as the inherent setting of the model.^28^

### 2.6 Quality Assurance and Quality Control

Recoveries and detection limits of all target compounds are listed in Table S3. Method blanks and matrix blanks were included during sample extraction to check for possible contamination. One solvent blank and one check standard (100 μg L^–1^) were injected after every 10 samples to check cross-contamination and reproducibility (RSD < 20%). No target compounds were detected in the method blanks, matrix blanks, and solvent blanks, indicating no background or cross-contamination during extraction and instrument analysis. Data are presented as mean ± standard deviation (SD). The data were analyzed using SPSS Statistics 28 (IBM Corp, Chicago, Illinois) and graphed using GraphPad Prism 9 (La Jolla, California). Statistical significance was generally derived by one-way analysis of variance (ANOVA) test, except that the calculated LC_50_ values were compared by the ratio test.^29^ The significance level was set at *p* < 0.05.

## 3 Results and Discussion

### 3.1 Acute Toxicity to **D. magna**

**Figure 1:**
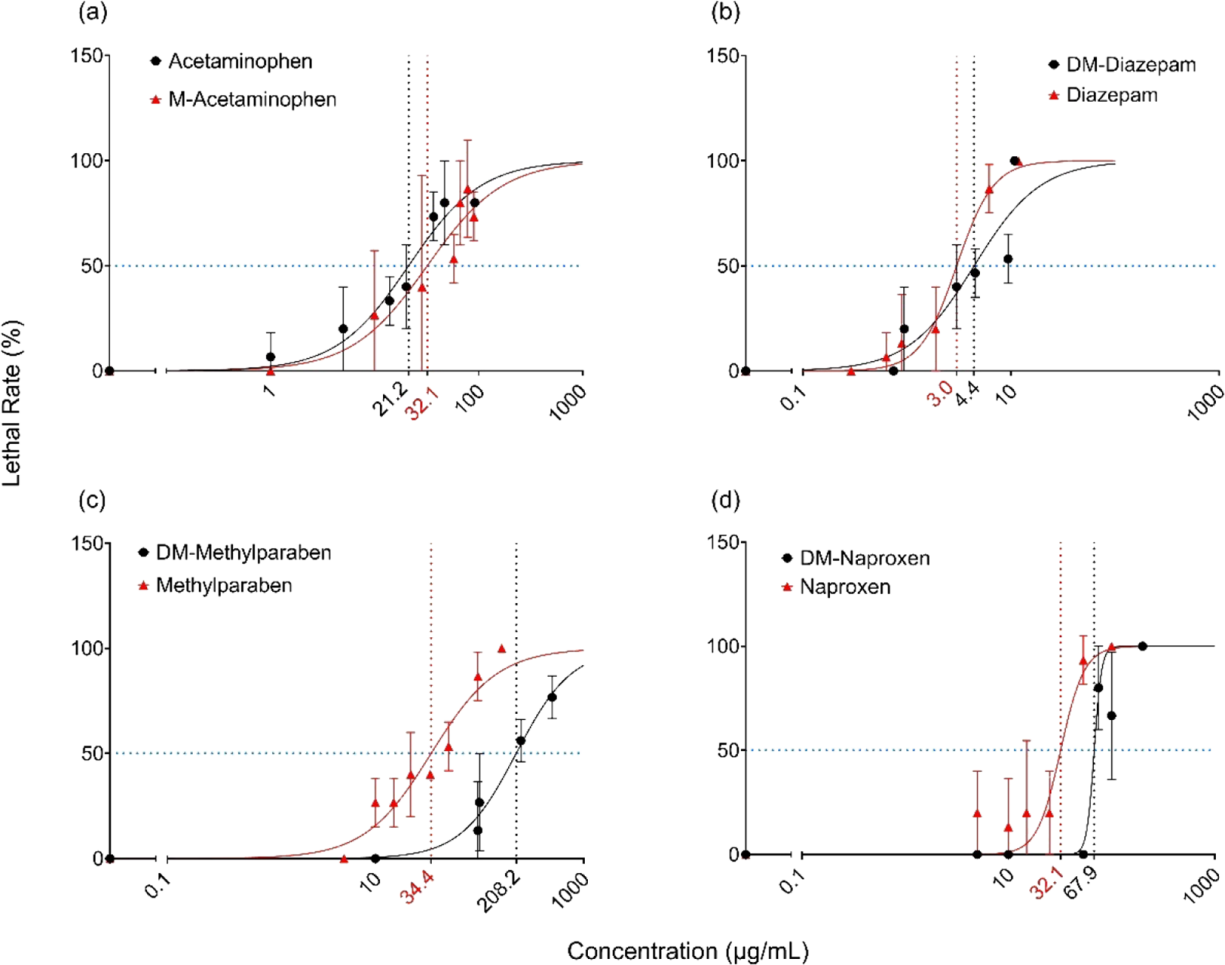
Concentration–response curves of (a) acetaminophen and M-acetaminophen, (b) DM-diazepam and diazepam, (c) DM-methylparaben and methylparaben, and (d) DM-naproxen and naproxen for **D. magna** over a 48 h acute exposure.

To evaluate the influence of methylation or demethylation on the acute toxicity of CECs, we exposed **D. magna** to a range of aqueous concentrations of individual CECs and their methylated or demethylated TPs for 48 h. The derived dose–response curves are plotted in Figure 1, along with the calculated LC_50_ values. Methylation or demethylation changed the acute toxicity of most CECs, and the influence was compound-specific. For example, methylation of acetaminophen caused a significant decrease in acute toxicity (*p* < 0.05), with LC_50_ increasing from 21.2 ± 2.4 mg L^–1^ for acetaminophen to 32.1 ± 5.7 mg L^–1^ for M-acetaminophen. In contrast, methylation had the opposite effect on the acute toxicity of DM-methylparaben and DM-naproxen. For example, DM-methylparaben was found to be significantly less toxic to **D. magna** than methylparaben (*p* < 0.05), with approximately an eightfold difference between their LC_50_ values. The LC_50_ of DM-naproxen was found to be 67.9 ± 6.0 mg L^–1^, which was significantly greater compared to naproxen (32.1 ± 4.9 mg L^–1^, *p* < 0.05). However, methylation and demethylation did not affect the acute toxicity of DM-diazepam and diazepam and there was no significant difference between their respective LC_50_ values.

To better understand how methylation and demethylation affect the acute toxicity of CECs, the derived log LC_50_ values of the target compounds are plotted against their corresponding log *D*_lipw_ values (Figure S2a). A significantly negative linear relationship was observed, indicating that as log *D*_lipw_ increased, LC_50_ for **D. magna** generally decreased, or the acute toxicity increased. Therefore, the changes in acute toxicity induced by methylation or demethylation of CECs may be partially attributed to changes in physicochemical properties, such as hydrophobicity. TPs with greater hydrophobicity tend to exhibit greater acute toxicity as compared to their parent form. After methylation, log *D*_lipw_ of DM-methylparaben and DM-naproxen increased from −1.62 to 1.93 and from −0.67 to −0.51, respectively, consistent with increases (seven- and twofold changes in LC50, respectively) in their toxicity. In a previous study, methylated diclofenac was found to be more toxic than diclofenac in **G. pulex** and **H. azteca**.^6^ Methylated derivatives of BPA were more toxic in zebrafish (**Danio rerio**) embryos.^21^ It must be noted that the influence of methylation or demethylation on properties such as log *D*_lipw_ depends on the overall molecular structure of the compound and the position of the methyl group. In this study, the presence of a methyl group did not appreciably change the predicted log *D*_lipw_ for DM-diazepam (3.16) and diazepam (3.06) (Table 1), which may explain their similar LC_50_ values for **D. magna** found in this study. Factors other than hydrophobicity may also regulate toxicity, such as mode of action, metabolism, and elimination. In this study, even though the methylated product of acetaminophen, *M*-acetaminophen, has a higher log *D*_lipw_ value (1.45) than that of acetaminophen (0.89), the derived LC50 was significantly larger for *M*-acetaminophen (32.1 ± 5.7 mg/L) than for acetaminophen (21.2 ± 2.4 mg/L) (Table 2). Likewise, in previous studies, the methylated ethers of TBBPA were found to be less toxic than TBBPA in earthworms after 72 h of exposure on filter paper (**Eisenia fetida**), or after a 14 day exposure in soil (**Metaphire guillelmi**), or in zebrafish embryos following aqueous exposure for 28 days.^22,30^

**Table 2:** Comparison between *in vivo* Experimental Results and *in silico* Predictions for Acute Toxicity, Dissipation, and Bioaccumulation of CECs and Their Methylated or Demethylated Counterparts

	LC_50_-48 h (mg L^–1^)				BCF (L kg^–1^, w.w.)				half-life (h)			
compound	*in vivo*	relative potency^a^	*in silico*	relative potency	*in vivo*	ratio^b^	*in silico*	ratio	*in vivo*^c^	ratio	*in silico*	ratio
acetaminophen	21.2 ± 2.4	1.0	27.1	1.0	0.3 ± 0.0	1.0	1.0	1.0	1.0	1.0	0.3	1.0
*M*-acetaminophen	32.1 ± 5.7	0.7	61.0	0.4	10.0 ± 0.0	33.3	1.5	1.5	0.4	0.4	1.6	5.3
*DM*-diazepam	4.4 ± 1.1	1.0	5.4	1.0	9.8 ± 0.3	1.0	44.5	1.0	5.8	1.0	12.5	1.0
diazepam	3.0 ± 0.3	1.5	4.2	1.3	9.0 ± 0.4	0.9	37.2	0.8	1.5	0.3	18.8	1.5
*DM*-methylparaben	225.6 ± 17.3	1.0	55.7	1.0	0.9 ± 0.5	1.0	2.8	1.0	2.2	1.0	1.4	1.0
methylparaben	34.4 ± 4.3	6.6	10.0	5.6	2.8 ± 0.2	3.1	3.9	1.4	2.7	1.2	0.5	0.4
*DM*-naproxen	67.9 ± 6.0	1.0	9.5	1.0	0	N/A	19.0	1.0	N/A	N/A	6.5	1.0
naproxen	32.1 ± 4.9	2.1	13.8	0.7	1.5 ± 0.6	N/A	84.2	4.4	4.3	N/A	41.8	6.4

a Relative potency was calculated as the ratio of LC_50_ of demethylated derivative over methylated derivative.

b Ratio was calculated as the value of methylated derivatives over that of the demethylated counterparts.

c *In vivo* half-life values were derived from the depuration rate in Table S4 in the SI.

Observations from this and other studies indicate that the effect of simple changes in the chemical structure caused by transformations such as methylation and demethylation on toxicity is complex and depends closely on the specific molecular structure of the compound undergoing the transformation. Different modes of action may contribute to the acute toxicity of CECs to **D. magna** after methylation or demethylation.^31^ CECs contain different functional groups that may have specific interactions with specific cellular components like enzymes or receptors in **D. magna**.^32^ However, the observed general correlation between hydrophobicity and acute toxicity in **D. magna** in this study implies that bioaccumulation driven by hydrophobicity was likely an important cause for the methylation or demethylation-induced changes in nontarget toxicity.

### 3.2 Bioaccumulation in **D. magna**

**Figure 2:**
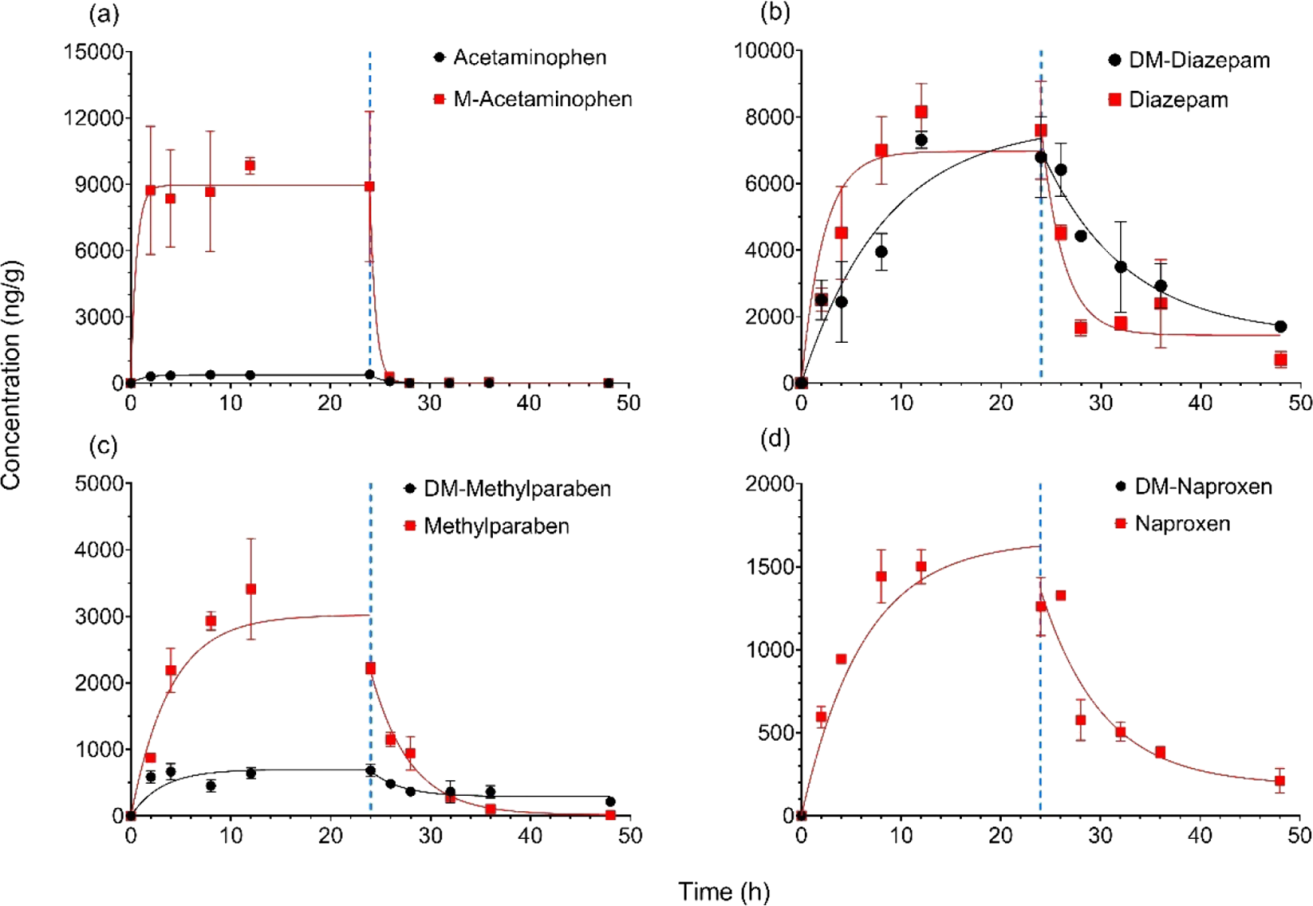
Bioaccumulation kinetics of the four pairs of CECs and their methylated/demethylated derivatives in **D. magna**: (a) acetaminophen and M-acetaminophen, (b) DM-diazepam and diazepam, (c) DM-methylparaben and methylparaben, and (d) DM-naproxen and naproxen.

To further understand the effect of methylation and demethylation on the acute toxicity to **D. magna**, the bioaccumulation of the CECs and their methylated or demethylated counterparts was measured in adult organisms. The concentrations of target compounds remained relatively constant in the aqueous media during the 24 h uptake phase, with RSDs ranging from 2.8 to 18.4% (Figure S1). Therefore, the mean measured concentrations of target compounds in the water phase were used as *C*_w_ to fit eqs 5 and 6 to derive the BCF value. The bioaccumulation kinetics of the target compounds are shown in Figure 2. The concentrations of CECs and their methylated or demethylated TPs generally showed an increasing trend at the beginning of the uptake phase and reached an apparent equilibrium in 24 h. Upon transfer of the exposed **D. magna** to clean AFW to initiate the depuration phase, the concentration of test compounds gradually declined over time. With the exception of diazepam, methylated derivatives consistently showed much higher concentrations in **D. magna** than in their demethylated counterparts. For example, after 2 h of exposure, the concentrations of acetaminophen and *M*-acetaminophen in **D. magna** were found at 308.7 ± 42.6 ng g^–1^ (w.w.) and 8730.7 ± 2900.9 ng g^–1^ (w.w.), respectively, a 28-fold difference (Figure 2a). This was consistent with the fact that methylated acetaminophen has a log *D*_lipw_ that is higher than that of acetaminophen (Table 1). In addition, at pH 8.5, acetaminophen was expected to be partially ionized in the aqueous media while *M*-acetaminophen should be completely in its neutral state (Table 1). Methylparaben also displayed a much higher accumulation (2216.3 ± 85.7 ng g^–1^, w.w.) than *DM*-methylparaben (682.7 ± 91.5 ng g^–1^, w.w.) in **D. magna** at the end of the uptake phase (24 h, Figure 2c). The threefold change also coincided with the difference in log *D*_lipw_ between *DM*-methylparaben (−1.62) and methylparaben (1.93) (Table 1). The level of *DM*-naproxen in **D. magna** was below the LOD, and therefore its bioaccumulation may be deemed negligible (Figure 2d). In contrast, significant accumulation of naproxen in **D. magna** was observed, again suggesting a pronounced effect of hydrophobicity induced by methylation. It is also likely that *DM*-naproxen was rapidly metabolized due to the presence of an exposed hydroxyl group (Table 1). The presence of the hydroxyl group in *DM*-naproxen may facilitate its conjugation with glucose in **D. magna**,^33,34^ contributing to its rapid metabolism and reduced bioaccumulation. Unlike the other three pairs, there was no significance in the bioaccumulation between *DM*-diazepam and diazepam in **D. magna** (Figure 2b), with 6792.5 ± 1215.8 ng g^–1^ (w.w.) and 7599.7 ± 1470.3 ng g^–1^ (w.w.) detected in **D. magna** after 24 h, respectively. This may be attributed to the fact that methylation or demethylation does not result in a great change in their physicochemical properties and that both compounds have similar log *K*_ow_ or log *D*_lipw_ values (Table 1).

The derived kinetic parameters of target compounds are given in Table S4. In general, the methylated derivative in each pair had a larger *k*_u_ than the corresponding demethylated counterpart. The dynamic BCF values, calculated as the ratio of *k*_u_ and *k*_d_, showed a strong correlation with the BCF values derived from the steady state (Figure S3, *R*^2^ = 0.98, *p* < 0.01), suggesting enhanced bioaccumulation for most methylated CECs. For example, the dynamic BCF of *M*-acetaminophen was 10.0 ± 0.0 in **D. magna**, which was significantly higher than the dynamic BCF of acetaminophen (0.3 ± 0.0). For *DM*-diazepam and diazepam, however, the BCF values in **D. magna** were not significantly different from each other, which again coincided with their generally similar physicochemical properties.

For aquatic organisms, increased bioaccumulation of contaminants is often attributed to a compound’s hydrophobicity, as bioaccumulation is driven by lipids in an organism and is positively related to hydrophobicity or log *K*_ow_ for neutral compounds.^16,20,26,35,36^ Increased bioaccumulation after methylation was previously observed for diclofenac in aquatic invertebrates. Bioaccumulation of methylated diclofenac was found to be 25–110-fold that of diclofenac in **H. azteca** and **G. pulex**.^6^ In this study, methylation generally increased the log *K*_ow_ of CECs, and further log *D*_ow_ and log *D*_lipw_, although the relative increases are specific to the individual compounds. The generally enhanced bioaccumulation in **D. magna** was also in agreement with the effect of methylation on CEC bioaccumulation in plants.^20^ Methylation of CECs could occur in natural water bodies due to the presence of methyl iodide,^7^ during wastewater treatment,^37^ and during biological transformations in soil,^17^ plants,^38^ and earthworms.^22^ Therefore, methylated derivatives of CECs may be prevalent in the environment and should be considered in a holistic risk assessment because of their different behaviors and biological activities such as increased bioaccumulation potentials.

### 3.3 Interconversion between CECs and Their Derivatives

**Figure 3:**
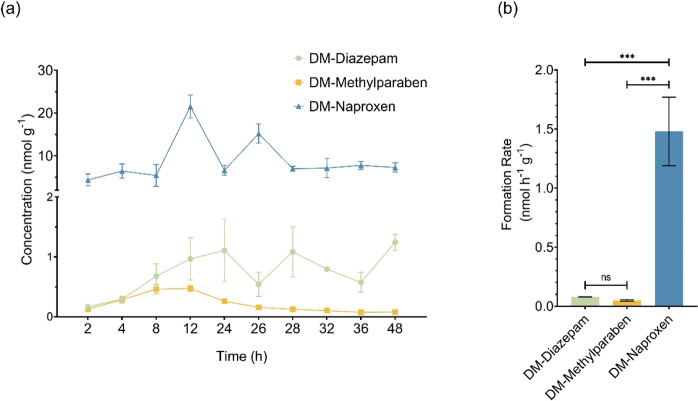
Formation of demethylated TPs in **D. magna** exposed to diazepam, methylparaben, or naproxen: (a) concentration kinetics over the exposure time period; (b) formation rates of demethylated TPs during the first 12 h period.

Biologically mediated transformations such as methylation and demethylation may also occur in organisms such as **D. magna** after their uptake of CECs, which may further influence their toxicity. Methylation and demethylation in **D. magna** were investigated after **D. magna** was exposed to the individual compounds. Methylation of the selected demethylated CECs was negligible as no methylated product was detected in **D. magna** after its exposure to the corresponding demethylated counterpart. However, demethylation of diazepam, methylparaben, and naproxen in **D. magna** was evident (Figure 3a) while acetaminophen was not detected in **D. magna** exposed to *M*-acetaminophen. The demethylation of methylparaben was limited, with a peak concentration of *DM*-methylparaben at 0.5 ± 0.0 nmol g^–1^ (w.w.) in **D. magna** after 12 h of exposure to 1 mg L^–1^ methylparaben. This represented only about 2.0% of the molar equivalent of methylparaben in **D. magna**. The demethylation of diazepam was found at similar levels, with *DM*-diazepam at 4.4% mol equivalent of diazepam. Interestingly, the molar equivalents of the demethylated derivatives continued to increase during the depuration phase, even though the overall concentration generally decreased over time. For example, the molar equivalents of *DM*-diazepam and *DM*-methylparaben reached 33.5 and 54.8% at the end of depuration, respectively. This may be attributed to the fact that demethylation continued during the depuration phase, which may have influenced the apparent depuration of these compounds (Table S4).

The demethylation of naproxen in **D. magna** was the most pronounced among the four methylated compounds, with *DM*-naproxen generally detected at levels higher than those of naproxen itself during both the uptake and depuration phases (Figure 3a). *DM*-Naproxen was formed quickly in **D. magna** after exposure to naproxen, with 21.5 ± 2.7 nmol g^–1^ (w.w.) after 12 h into the uptake phase, which was significantly higher than that of the parent naproxen (6.5 ± 0.4 nmol g^–1^, w.w.). Similar to the case for *DM*-diazepam and *DM*-methylparaben, the molar equivalent of *DM*-naproxen also continued to increase during the depuration phase. At the end of depuration, *DM*-naproxen accounted for approximately 88.9% of the total naproxen and *DM*-naproxen residues in **D. magna**. The high proportion of *DM*-naproxen in **D. magna** also suggested that demethylation was the primary metabolism pathway of naproxen in **D. magna**.

To better understand the demethylation of CECs in **D. magna**, the formation rates of *DM*-diazepam, *DM*-methylparaben, and *DM*-naproxen were estimated (Figure 3b) by simulating their formation over the initial 12 h period, during which good linear relationships between their formation and time were present (Figure S4). Formation rates showed no significant differences between those of *DM*-diazepam and *DM*-methylparaben. However, the formation rate of *DM*-naproxen (1.5 ± 0.3 nmol g^–1^ h^–1^) was significantly greater than that of *DM*-diazepam or DM-methylparaben. Based on their respective chemical structures (Table 1), the demethylation of diazepam and naproxen appears to differ slightly from that of methylparaben. While the demethylation of methylparaben involves the removal of a methyl group from a carboxyl group, which may be catalyzed by carboxylesterases,^39,40^ CYP450s,^41^ or through nonenzymatic hydrolysis,^40,42^ the demethylation of *M*-acetaminophen, diazepam, and naproxen reflects the removal of a methyl group from an amide or hydroxyl group, which likely is catalyzed mainly by CYP450s.^19,43^ Previous studies showed that carboxylesterases play a more important role in drug metabolism in invertebrates due to the lower activity of CYP450s.^44^ The more significant demethylation observed for naproxen in comparison to that of methylparaben suggests that CYP450s may also play an important role in the metabolism of such substrates in aquatic invertebrates. The observed significant differences in the demethylation rates of diazepam and naproxen imply that CYP450s in aquatic invertebrates like **D. magna** may exhibit different levels of activity toward different CECs. Furthermore, naproxen was fully ionized and highly hydrophilic compared to diazepam and methylparaben (Table 1). It is possible that ion trapping effects may occur, and these effects could be particularly significant for naproxen, contributing to its rapid demethylation in **D. magna**.

### 3.4 *In silico* Predictions

QSAR models are often employed for predicting the environmental fate of man-made chemicals for which experimental data are not available, enabling a preliminary assessment of their environmental risks. In this study, several environmental parameters of CECs and their methylated or demethylated derivatives were predicted using QSAR models and the predicted values were further compared against the experimentally derived data (Table 2). The LC_50_ values computed by the T.E.S.T. software aligned well with experimental data for the neutral compounds, including acetaminophen, *M*-acetaminophen, *DM*-diazepam, and diazepam (*R*^2^ = 0.95, *p* < 0.05). For example, *in vivo* LC_50_ values of *DM*-diazepam and diazepam in **D. magna** were 4.4 ± 1.1 and 3.0 ± 0.3 mg L^–1^, respectively, while the *in silico* values were 5.4 and 4.2 mg L^–1^ for *DM*-diazepam and diazepam, respectively. However, for the partially ionized compound methylparaben and the fully ionized compounds *DM*-methylparaben, *DM*-naproxen, and naproxen, *in silico* predicted acute toxicity was greater as compared to the *in vivo* results. For example, the predicted LC_50_ of *DM*-naproxen in **D. magna** was 9.5 mg L^–1^, which was much lower than the experimental value of 67.9 ± 6.0 mg L^–1^. However, the relative potency, as determined by dividing the LC_50_ of the demethylated derivative in each pair by that of its methylated counterpart,^26^ suggested that the influence of methylation or demethylation on the acute toxicity of CECs in **D. magna** may be adequately predicted using *in silico* methods (*R*^2^ = 0.94, *p* < 0.05).

*In silico* BCF values were obtained for lower trophic fish, in lieu of **D. magna**, using the BCFBAF model in the U.S. EPA’s EPI suite software (v 4.11). Since the derived BCF values could not be directly compared with the *in vivo* BCF values obtained for **D. magna** in this study, a relative bioaccumulation ratio was calculated by dividing the BCF of the demethylated derivative in each pair by that of its methylated counterpart. The tendency of bioaccumulation after methylation or demethylation of CECs predicted by the QSAR models generally agreed with the *in vivo* results, although the correlation was not statistically significant, likely due to the small sample size. The *in silico* predictions in this study showed that QSARs may underestimate the increases in the bioaccumulation potential of CECs from methylation. For instance, the BCF of acetaminophen rose by approximately 33-fold in **D. magna** after methylation while the *in silico* approach projected only a 50% increase in small fish.

*In vivo* half-lives of the test compounds were derived from the depuration rate (*k*_d_, h^–1^) during the 24 h depuration phase in **D. magna**. The *in silico* half-life was estimated from the primary biotransformation rate in fish and normalized to a 10 g fish at 15 °C based on the inherent characteristics of the QSAR model.^27,28^ Similar to the BCF values, *in vivo* and *in silico* half-lives could not be compared directly between the different organisms. Hence, the relative persistence of test compounds was calculated for evaluation. As shown in Table 2, *in silico* predictions suggest that methylation prolongs the persistence of CECs in fish. This was in contrast to the *in vivo* results in **D. magna**, which showed that methylation generally shortened the persistence of CECs. As mentioned above, methylated CECs generally accumulated faster with a larger *k*_u_ value during the uptake phase but dissipated rapidly during the depuration process. Considering that biota residing in wastewater effluent-dominated streams often experience pseudopersistent exposure to CECs due to the constant discharge of effluents from WWTPs, uptake rates may be more important in regulating the accumulation of CECs in aquatic organisms dwelling in the impacted system. The prolonged biotransformation half-lives of methylated CECs should be validated under the field conditions.

Overall, *in silico* predictions and experimental measurements were in agreement for the influences introduced by methylation or demethylation. This highlights the feasibility of incorporating QSAR models to evaluate the potential influence of common transformations such as methylation and demethylation on the environmental risks of CECs to nontarget organisms in impacted ecosystems.

### 3.5 Conclusions and Environmental Implications

Simple changes in the chemical structure caused by transformations such as methylation and demethylation contribute to the proliferation of the numbers of CECs and diverse structures in environmental compartments impacted by, e.g., wastewater effluent.^10,13,15,16^ This study showed that these transformations can alter the physicochemical properties of CECs, resulting in changes in their environmental processes, such as bioaccumulation and acute toxicity, in aquatic organisms. These transformations of man-made chemicals may also take place within a nontarget organism after their uptake from the ambient environment. Certain transformations, like methylation, likely lead to enhanced bioaccumulation and increased toxicity in nontarget organisms. For example, methylparaben showed greater acute toxicity to **D. magna** and higher bioaccumulation potential than *DM*-methylparaben. These changes may be attributed to the increased hydrophobicity after methylation. Although not considered in this study, halogenation of man-made chemicals, such as gemfibrozil, 4-nonylphenol, and naproxen, during the disinfection process in WWTPs has also been reported and the halogenated products generally exhibited increased bioaccumulation and toxicity to aquatic invertebrates.^26,45,46^ Due to the presence of numerous CECs in sources such as wastewater effluents and sediments, the coexistence of various TPs presents an additional challenge in addressing the overall environmental risks of man-made chemicals.

The ecotoxicological data for the compounds examined in this study, especially the methylated or demethylated TPs, were not available. Therefore, the acute toxicity test was used to provide an initial understanding of the potential effects of such transformations. The concentrations used in the acute toxicity test were likely above environmentally relevant levels. Further research should consider sublethal effects under environmentally relevant conditions. Previous studies suggested that BCFs may be greater at lower exposure concentrations.^25^ Therefore, the effect of methylation or demethylation on the bioaccumulation of CECs may be more pronounced than what was observed in this study. The environmental occurrence and concentration of methylated or demethylated TPs are largely unknown for most CECs. Further research into the occurrence of TPs in different environmental compartments is needed to gain knowledge about the realistic exposure levels and refine the risk assessment.

A major challenge in comprehensively assessing environmental risks is the sheer number of CECs and their TPs. It is unrealistic to experimentally evaluate transformation-induced changes in the environmental behaviors and toxicological profiles for all CECs.^47^ The incorporation of well-established QSAR models to predict essential chemical properties and environmental risk markers, such as hydrophobicity and lipophilicity, bioaccumulation potential, and acute toxicity, may help prioritize TPs with increased biological activities.^48−50^ This approach can be used to more effectively direct future research efforts to better understand the environmental significance of common transformation reactions for CECs.

## Supporting Information Available

(Text 1) Calculation of physicochemical properties; (Tables S1 and S2) additional information on analytical methods; (Table S3) parameters form bioaccumulation kinetics; and (Figures S1–S4) additional figures showing chemical concentrations in media, relationships between chemical properties and bioaccumulation, and formation of demethylated products as a function of time (PDF)
