# Supplementary material for: Methylation and Demethylation of Emerging Contaminants Changed Bioaccumulation and Acute Toxicity in Daphnia magna
Source: Environ Sci Technol. 2023 Sep 28;57(40):15213–22. doi: 10.1021/acs.est.3c03242 (PMC10569044; doi:10.1021/acs.est.3c03242)
Supplement: Supplementary file 1 — es3c03242_si_001.pdf [file es3c03242_si_001.pdf]

Methylation and demethylation of emerging contaminants changed bioaccumulation and acute toxicity in *Daphnia magna*

Text 1. Calculation of physicochemical property parameters

The pH of the artificial freshwater environment for *D. magna* was measured as 8.10 ± 0.10. The fraction of neutral species (f_n) for the test compounds was calculated as the following equation:[1–3]

$$f_n = \frac{1}{1 + 10^{i(pH - pK_a)}} \tag{1}$$

where i is 1 for acids and -1 for bases. The pH-adjusted octanol-water coefficient log D_{ow} was estimated as:

$$logD_{ow} = logK_{ow} + logf_n \tag{2}$$

The pH-adjusted liposome-water partition coefficient (log D_{lipw}) was calculated using the following equation:[4]

$$logD_{lipw} = 0.9 * logD_{ow} + 0.52 \tag{3}$$

The physicochemical parameters of all target compounds are summarized in Table 1. The relationship between log D_{lipw} of the target compounds and their corresponding acute toxicity (LC_{50}) and bioconcentration factor (BCF) was evaluated through linear regression analysis (Figure S2).

Table S1. MRM transitions for test compounds on UPLC-MS/MS

Compound	MRM (m/z)			
	Quantification	CV/CE*	Qualification	CV/CE
ESI+				
Acetaminophen	151.97 > 109.99	38/22		
M-Acetaminophen	166.03 > 124.07	38/22	166.03 > 92.74	38/24
d4-Acetaminophen	156.03 > 113.99	40/12	156.03 > 96.75	40/22
DM-diazepam	271.03 > 139.99	56/28	271.03 > 165.03	56/28
Diazepam	285.03 > 154.02	56/26	285.03 > 193.09	56/32
d5-Diazepam	290.10 > 198.07	54/34	290. 10 > 154.11	54/26
ESI-				
DM-Methylparaben	137.09 > 93.08	34/15		
Methylparaben	151.05 > 92.03	38/20	151.05 > 136.00	38/14
d4-Methylparaben	155.05 > 96.05	36/20	155.05 > 140.01	36/14
DM-Naproxen	215.15 > 171.15	21/6	215.15 > 169.15	21/28
Naproxen	229.15 > 185.15	17/8	229.15 > 170.15	17/16
d3-Naproxen	232.18 > 188.10	14/5	232.18 > 173.14	14/18

*CV-cone voltage (kV), CE-collision energy (eV).

Table S2. Recoveries and limits of quantification (LOQ) of test compounds.

Compound	LOQ (ng/mL)	Recovery (%)	
		D. magna	AFW
Acetaminophen	0.5	84.6 ± 5.3	87.4 ± 1.8
M-acetaminophen	0.2	62.0 ± 5.7	99.6 ± 2.3
DM-diazepam	0.2	103.5 ± 11.0	105.0 ± 2.7
Diazepam	0.25	128.4 ± 3.2	94.3 ± 2.3
DM-methylparaben	3.0	51.9 ± 7.3	72.6 ± 12.3
Methylparaben	1.5	95.8 ± 3.2	116.8 ± 1.8
DM-naproxen	3.0	60.9 ± 12.6	119.8 ± 2.2
Naproxen	2.0	127.6 ± 1.1	106.4 ± 1.3

Table S3. Bioaccumulation kinetic parameters of the target CECs in *D. magna*.

Compound	k_u (L kg^{-1} h^{-1})	R^2	k_d (h^{-1})	R^2
Acetaminophen	0.2 ± 0.0	0.991	0.8 ± 0.0	1.000
M-Acetaminophen	17.3 ± 0.4	0.982	1.7 ± 0.0	1.000
DM-Diazepam	1.2 ± 0.1	0.900	0.1 ± 0.0	0.968
Diazepam	4.1 ± 0.3	0.879	0.4 ± 0.2	0.926
DM-Methylparaben	0.3 ± 0.0	0.620	0.3 ± 0.1	0.895
Methylparaben	0.7 ± 0.1	0.855	0.2 ± 0.0	0.986
DM-Naproxen	_	_	_	_
Naproxen	0.2 ± 0.0	0.855	0.2 ± 0.1	0.868

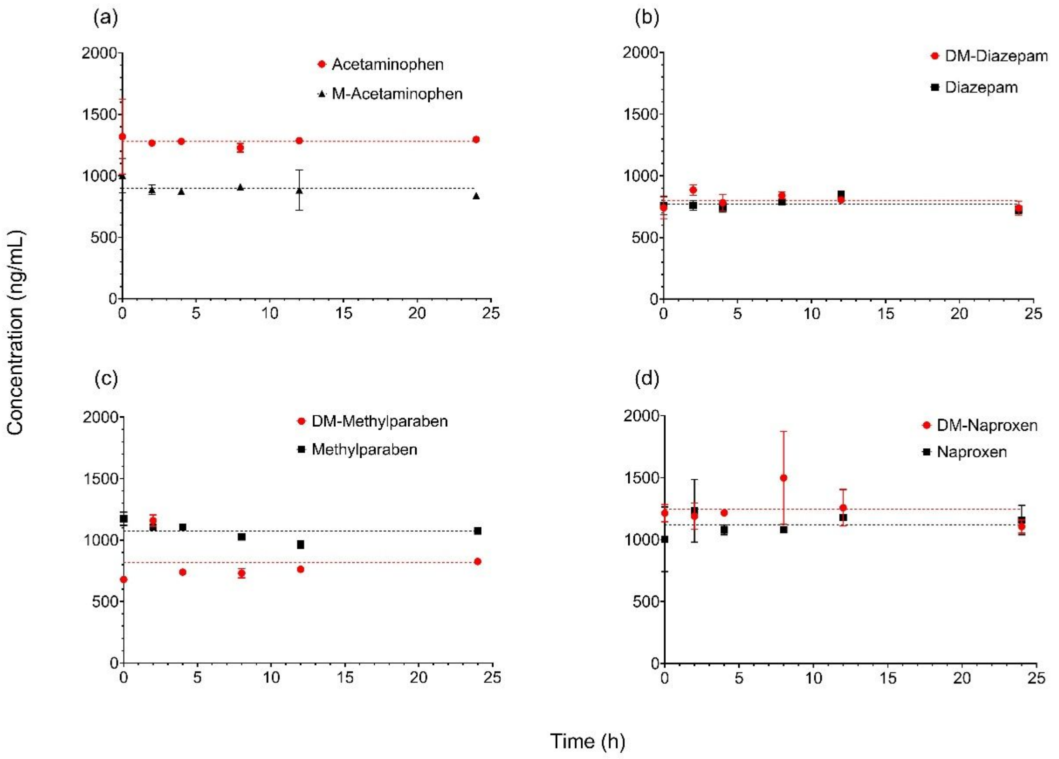

Figure S1. Concentrations of test compounds in the artificial freshwater during the uptake phase.

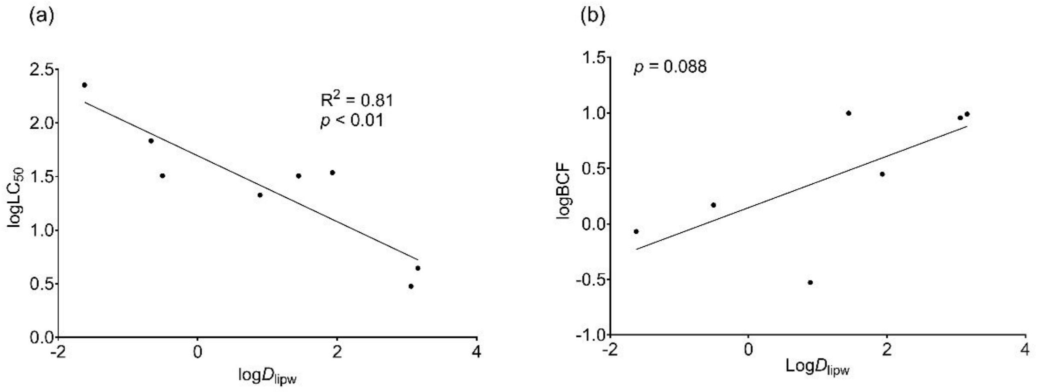

Figure S2. Relationships between $\log D_{\mathrm{lipw}}$ and (a) $\log LC_{50}$ and (b) logBCF.

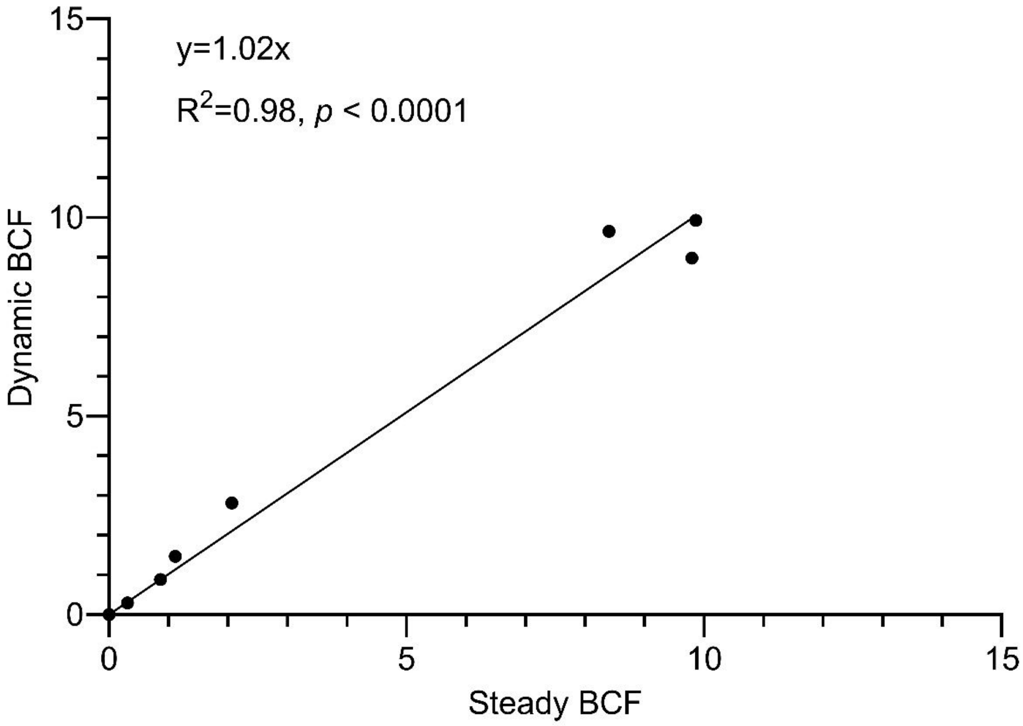

Figure S3. The correlation between steady state BCF and dynamic BCF in *D. magna*

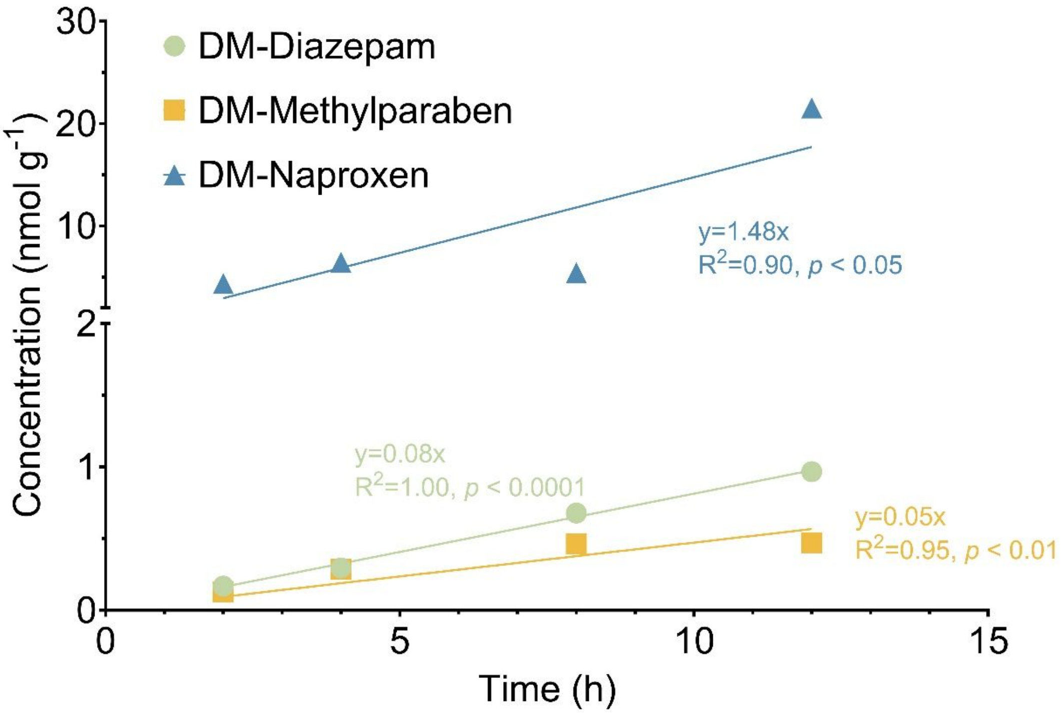

Figure S4. Linear correlations between the concentration of the formed demethylated derivatives in *D. magna* and the exposure time to the corresponding methylated parent compounds

References

(1) Wu, X.; Ernst, F.; Conkle, J. L.; Gan, J. Comparative Uptake and Translocation of Pharmaceutical and Personal Care Products (PPCPs) by Common Vegetables. *Environ Int* **2013**, *60*, 15–22. https://doi.org/10.1016/j.envint.2013.07.015.

(2) Xiong, Y.; Shi, Q.; Sy, N. D.; Dennis, N. M.; Schlenk, D.; Gan, J. Influence of Methylation and Demethylation on Plant Uptake of Emerging Contaminants. *Environ Int* **2022**, *170*, 107612. https://doi.org/10.1016/j.envint.2022.107612.

(3) Trapp, S. Bioaccumulation of Polar and Ionizable Compounds in Plants. In *Ecotoxicology Modeling*; Devillers, J., Ed.; Springer US: Boston, MA, 2009; pp 299–353. https://doi.org/10.1007/978-1-4419-0197-2_11.

(4) Ding, J.; Lu, G.; Liu, J.; Yang, H.; Li, Y. Uptake, Depuration, and Bioconcentration of Two Pharmaceuticals, Roxithromycin and Propranolol, in Daphnia Magna. *Ecotoxicol Environ Saf* **2016**, *126*, 85–93. https://doi.org/10.1016/j.ecoenv.2015.12.020.
